# Genomic Variability of Monkeypox Virus among Humans, Democratic Republic of the Congo

**DOI:** 10.3201/eid2002.130118

**Published:** 2014-02

**Authors:** Jeffrey R. Kugelman, Sara C. Johnston, Prime M. Mulembakani, Neville Kisalu, Michael S. Lee, Galina Koroleva, Sarah E. McCarthy, Marie C. Gestole, Nathan D. Wolfe, Joseph N. Fair, Bradley S. Schneider, Linda L. Wright, John Huggins, Chris A. Whitehouse, Emile Okitolonda Wemakoy, Jean Jacques Muyembe-Tamfum, Lisa E. Hensley, Gustavo F. Palacios, Anne W. Rimoin

**Affiliations:** United States Army Medical Research Institute of Infectious Diseases, Fort Detrick, Maryland, USA (J.R. Kugelman, S.C. Johnston, M.S. Lee, G. Koroleva, S.E. McCarthy, M.C. Gestole, J. Huggins, C.A. Whitehouse, G.F. Palacios);; Kinshasa School of Public Health, Kinshasa, Democratic Republic of the Congo (P.M. Mulembakani, E.O. Wemakoy);; University of California, Los Angeles, California, USA (N. Kisalu, A.W. Rimoin);; Global Viral Forecasting (now known as Metabiota), San Francisco, California, USA (N.D. Wolfe, J.N, Fair, B.S. Schneider);; The Eunice Kennedy Shriver National Institute of Child Health and Human Development, Bethesda, Maryland, USA (L.L. Wright);; National Institute of Biomedical Research, Kinshasa (J.J. Muyembe-Tamfum);; US Food and Drug Administration, Silver Spring, Maryland, USA (L.E. Hensley)

**Keywords:** Monkeypox virus, genomic diversity, emerging infectious disease, genomic reduction, gene loss, Democratic Republic of the Congo, viruses

## Abstract

Monkeypox virus is a zoonotic virus endemic to Central Africa. Although active disease surveillance has assessed monkeypox disease prevalence and geographic range, information about virus diversity is lacking. We therefore assessed genome diversity of viruses in 60 samples obtained from humans with primary and secondary cases of infection from 2005 through 2007. We detected 4 distinct lineages and a deletion that resulted in gene loss in 10 (16.7%) samples and that seemed to correlate with human-to-human transmission (p = 0.0544). The data suggest a high frequency of spillover events from the pool of viruses in nonhuman animals, active selection through genomic destabilization and gene loss, and increased disease transmissibility and severity. The potential for accelerated adaptation to humans should be monitored through improved surveillance.

## Abstract 2

Health authorities should be vigilant for this rapidly evolving virus.

Viruses in the family *Poxviridae,* genus *Orthopoxvirus*, consist of numerous pathogens known to infect humans, including variola virus (VARV), monkeypox virus (MPXV), cowpox virus (CPXV), and vaccinia virus (VACV). Genomes of these viruses are ≈200 kb long, have highly conserved central regions coding for replication and assembly machinery, and have more variable terminal ends that contain genes involved in host range determination and pathogenesis (*1*).

Although orthopoxviruses are antigenically and genetically similar, they have diverse host range and virulence properties (*1*–*6*). Comparative genomics studies have shown that the evolution of orthopoxviruses is ongoing and can be driven by selective pressure from a host species (*4*,*7*). It has been postulated that progressive gene loss, primarily at the terminal ends of the genome, has been a driving force behind the evolution of these viruses (*7*). CPXV, which causes only mild infection in humans, contains the largest genome of all sequenced orthopoxviruses (≈220 kb), encodes 223 open reading frames (ORFs), and has a broad host range that includes rodents, humans, felids, bovids, and voles (*7*–*10*). Conversely, VARV, the causative agent of smallpox, is highly pathogenic (case-fatality rate ≈30%) (*11*), has the smallest genome of all naturally occurring orthopoxviruses (≈186 kb), is predicted to encode 20% fewer functional proteins than CPXV, and has a host range restricted to humans (*7*).

Similar to VARV, MPXV is a virulent orthopoxvirus that causes high levels of illness and death (case-fatality rate ≈10%) (*12*–*15*). Unlike natural VARV infections, which were declared eradicated in 1979, MPXV infections occur naturally in MPXV-endemic regions of Africa, such as the Democratic Republic of the Congo (DRC) and Republic of the Congo. Human exposure to animal reservoirs, including squirrels of the genera *Funisciurus* and *Heliosciurus*, poses high risk for MPXV infection (*16*–*19*). Recent reports suggest that human-to-human transmission of MPXV is increasing (*20*,*21*). In 2003 in the DRC, 7 generations of uninterrupted spread among humans were reported (*21*). Increasing susceptibility coincident with decreasing herd immunity is expected to profoundly increase the introduction and spread of MPXV among humans. In 2003, an outbreak of monkeypox in the midwestern United States revealed the propensity for transmission to MPXV-naive populations (*22*). The strain of MPXV responsible belonged to the Western African clade; these strains tend to be less pathogenic than the Central African strains that circulate in the DRC (*2*).

MPXV is a linear DNA genome of ≈197 kb and contains ≈190 nonoverlapping ORFs >180 nt long (*1*,*7*,*26*). Like all orthopoxviruses, the central coding region sequence (CRS) at MPXV nucleotide positions ≈56000–120000 is highly conserved and flanked by variable ends that contain inverted terminal repeats (ITRs) (*1*). VACV homologs to genes found in the terminal ends of the MPXV genome are predominantly involved in immunomodulation, and most are either predicted or known to influence host range determination and pathogenicity (*27*). Unlike VARV, which lacks ORFs in the ITR region, MPXV contains at least 4 ORFs in the ITR region (*7*,*26*).

The evolution of short-read, high-throughput sequencing technologies has made genomic surveys of large populations readily attainable (*23*,*24*). It has been suggested that the progressive loss of genes that were not essential for pathogenesis in humans is the mechanism that led to the emergence of a highly adapted virus that causes serious disease and is capable of efficient and rapid human-to-human transmission (*7*). A recent study demonstrated that gene copy number variation might be a crucial factor for modulating virus fitness (*25*). In the study reported here, we investigated the genomic plasticity of MPXV strains obtained from patients in the DRC during active surveillance for monkeypox disease (*20*).

## Materials and Methods

### Sample Selection

 From November 2005 through November 2007, a total of 760 cases of human monkeypox in the Sankuru District, DRC, were identified by active disease surveillance and confirmed by quantitative real-time PCR (*20*). From these, we selected 60 cases on the basis of 3 epidemiologic measures: severity, transmission, and geography. The samples chosen represented the entire set of samples and included samples from every category of severity and transmission observed and every geographic location sampled. Of the 60 cases, we sequenced 29 representative scab or vesicle fluid samples from patients whose samples met the criteria for concentration and quality of genetic material (>500 ng total DNA and >1.8 260/230 and 260/280 absorbance ratio). We used the Genome Analyzer IIx and MiSeq sequencers (Illumina, Inc., San Diego, CA, USA) to conduct 76-bp or 100-bp, paired-end sequencing. To avoid laboratory-induced mutation events, we sequenced these samples directly from scab or vesicle material without virus isolation or propagation.

### Sample Collection and Processing

 Techniques for sample collection, processing, nucleotide isolation, and real-time PCR have been described (*20*,*28*,*29*). The World Health Organization scoring system used during the smallpox eradication program was used to generate severity designations: presence of <25 lesions on the body was considered mild disease; 25–99 lesions, moderate; 100–250 lesions, severe, and >250 lesions, serious. Libraries with an average insert size of ≈300 bp were processed by using the TruSeq (Illumina, Inc.) sequencing kit version 5 and sequenced for either 76 or 100 cycles from each end on an Illumina Genome Analyzer IIx or MiSeq instrument.

### Genome Assembly

 All raw Illumina sequence data were processed through an in-house–developed filtering program that removes or trims reads with known artifacts. Specifically, all reads containing adaptor sequence, low-complexity reads, or PCR duplicates were removed or trimmed. Host read exclusion was performed by removing reads that aligned to the human genome (*Homo sapiens* chromosome 10, GRCh37 primary reference assembly) by using the CASAVA pipeline 1.8 (Illumina, Inc.) with default parameters. De novo assemblies were performed by using SeqMan Pro NGen (DNAStar, Inc., Madison, WI, USA) with default parameters. Genome size was set to 200 kb. The optimal *k-*mer was determined by using an iterative assembly process. We closed gaps in coverage and resolved large repeats by using Sanger sequencing for the 23 genomes described in this work. Sequences were submitted to GenBank with accession nos. JX878407–JX878429.

### Phylogenetic Analysis

**Figure 1:**
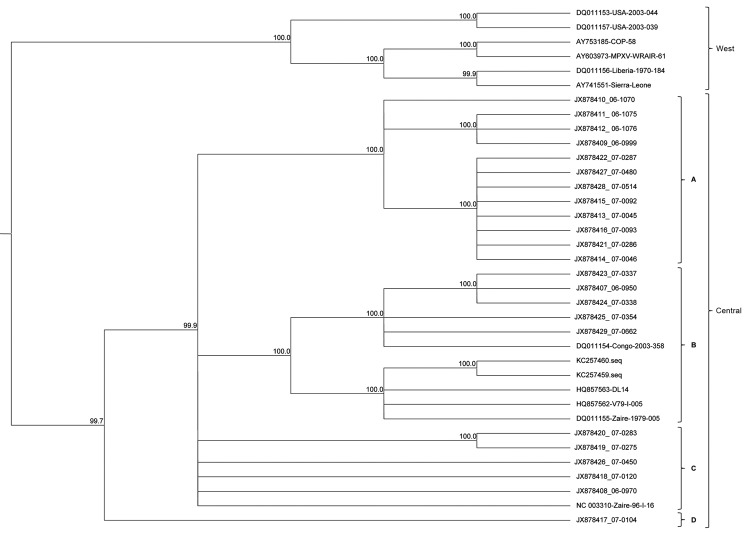
Phylogenetic analysis of whole-genome direct sequencing. Evolutionary relationships between sequenced samples and archived monkeypox virus (MPXV) sequences were determined for the central coding region sequence (MPXV nucleotide positions ≈56000–120000). A cladogram representing the topology of an unrooted Bayesian phylogenetic reconstruction is shown for samples identified by sample number and/or GenBank accession number. The Central and Western African clades and the 4 distinct lineages are indicated. Confidence values for branching events were computed by Markov chain Monte Carlo convergence. Numbers at nodes represent Bayesian posterior probabilities computed by using MrBayes 3.1.2 (*30*).

 We conducted the phylogenetic analysis by using methods identical to those reported for VARV diversity (*4*). In brief, we aligned the CRS (MPXV nucleotide positions ≈56000–120000) of samples with sufficient sequence information with 11 MXPV reference sequences available through the National Center for Biotechnology Information (www.ncbi.nlm.nih.gov/), GenBank nos. KC257459, KC257460, DQ011154, DQ011155, HQ857562, HQ857563, NC_003310, AY603973, AY753185, AY741551, DQ011156, DQ011153, and DQ011157 in SeqMan Pro NGen (DNAStar, Inc.). Manual editing of gaps and ambiguous base calls of the alignment were performed to include the draft genomes and provide a more robust phylogenetic narrative. The resulting alignment was analyzed by using Bayesian methods of phylogenetic reconstruction and the GTR+Γ evolutionary model. MrBayes version 3.1.2 (*30*) was run by using 1,000,000 generations with 4 parallel runs. Markov chain Monte Carlo convergence was assessed by checking the average standard deviation of split frequencies (<0.01) during >10,000 generations. Figure 1 shows the unrooted tree based on alignment of the 11 MPXV reference sequences and the viral genomes from the 23 clinical samples in this study. 

### PCR Screening 

 All primers were developed by using Primer-BLAST (www.ncbi.nlm.nih.gov/tools/primer-blast/) with default parameters. This software uses Primer3 to design the primers and then BLAST to determine specificity. We designed qualitative PCRs to detect the left and right short tandem repeats (STRs) at MPXV nucleotide positions 72–486 and 196710–197124, a region spanning the 625-bp deletion between bases 189820 and 190444 in the right ITR, and a 410-bp region of the CRS that contains 4 single-nucleotide polymorphism locations positively typing the lineages described in this article between nucleotide positions 89273 and 89944. All PCRs were run by using standard methods and optimized annealing temperatures of 55°C through 65°C. The PCR product Sanger sequences were aligned to the reference in SeqMan Pro software (DNAStar) for validation by using default parameters. Primer sequences are available on request.

## Results

 Referenced and de novo alignments were performed by using SeqMan Pro NGen to determine the scaffold of the viral genome. Genomes were finished by using PCR and traditional dideoxynucleotide sequencing (Sanger) to resolve gaps and repetitive and low coverage areas. We obtained 23 MPXV genomes, expanding the available completed genomes for Central African clade MPXV strains by ≈6-fold. To assess the potential genomic diversity of MPXV strains circulating in the DRC, we performed a phylogenetic analysis of the CRS. The resulting cladogram suggested the emergence of 4 distinct lineages within the Central African clade.

### Genomic Reduction

**Figure 2:**
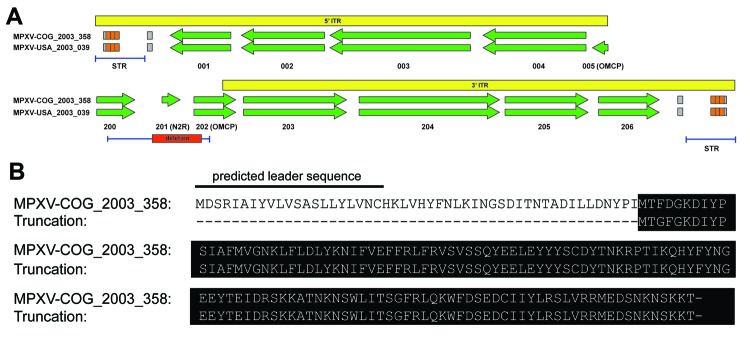
Truncation of OMCP. A) Whole-genome deep sequencing revealed a 625-bp deletion directly upstream of the right ITR (red box), which completely removed MPV-Z-N2R (locus 201) and truncated OMCP (MPV-Z-N3R, locus 202). A Western African clade virus, MPXV-USA_2003_039, is shown for comparison with OMCP and N2R copy number. PCR amplification regions for the large deletion and the STRs (right and left) are indicated by the blue bar below each ITR diagram. The yellow box represents the ITR region, and all open reading frames are identified by their MPXV-COG_2003_358 locus number with genes of interest in parenthesis. B) Nucleotide alignment of the large deletion with MPXV-COG_2003_358 reference sequence. The truncated protein created by the deletion is indicated by the black background. ITR, inverted terminal repeat; MPXV, monkeypox virus; COG, Congo; STR, short terminal repeat; OMCP, orthopoxvirus major histocompatibility complex class I–like protein.

 Among the MPXV alignments, we detected a polymorphism in the noncoding region of the ITR with 12 variants. Of the 23 (17.4%) complete genome sequences, 4 showed substantial genomic instability directly upstream of the right ITR. This subset of samples contained a 625-bp deletion between bases 189820 and 190444 (genome positions based on MPXV-COG_2003_358). This deletion completely removes MPV-Z-N2R and eliminates the first 103 bp of the orthopoxvirus major histocompatibility complex class I–like protein (OMCP, MPV-Z-N3R) (Figure 2, panel A). The function of the protein expressed from MPV-Z-N2R is unknown, and no homologous gene is in the VARV or West African MPXV genomes. OMCP is a secreted protein that binds to NKG2D and interferes with NKG2D-mediated cell killing by natural killer cells (*31*). A conventional PCR created to characterize the deletion enabled us to detect this deletion in 6 additional genomes (Table) We found no evidence of a mixed population within the sequence information for the isolates containing the deletion or in the gel electrophoresis of the Amplicon Sanger sequencing products for samples tested (data not shown).

**Table:** Consolidated patient metadata, phylogenetic lineage, STR, and deletion analysis data for patients with monkeypox virus infection, Democratic Republic of the Congo, November 2005–November 2007*

Patient	Age, y, Sex	GenBank no.	Deletion	STR	Lineage	Date	Geographic zone	Disease severity	Transmission
06–1105	3.8, M	NA	−	1	A	2006 Dec 8	Vangakete	Moderate	NA
07–0663	4.8, F	NA	−	1	A	2007 Sep 11	Lomela	Moderate	Primary
07–0748	13.0, M	NA	−	1	A	2007 Nov 13	Lomela	Severe	Primary
07–0107	6.0, M	NA	−	2	A	2007 Jan 16	Lodja	Severe	Secondary
07–0110	24.0, F	NA	−	2	A	2007 Jan 16	Lodja	Moderate	Secondary
07–0131	14.0, M	NA	−	2	A	2007 Jan 28	Lodja	Moderate	Secondary
07–0132	12.0, F	NA	−	2	A	2007 Jan 28	Lodja	Moderate	Secondary
07–0133	0.3, M	NA	−	2	A	2007 Jan 28	Lodja	Mild	Secondary
07–0641	32.0, M	NA	−	2	A	2007 Jun 5	Kole	Severe	Primary
07–0749	11.0, M	NA	−	3	A	2007 Nov 17	Lomela	Moderate	Primary
07–0125	14.0, M	NA	−	4	A	2007 Jan 14	Lomela	Moderate	Primary
07–0130	2.1, M	NA	−	4	A	2007 Jan 20	Lomela	Moderate	Primary
07–0287	13.0, M	JX878422	−	4	A†	2007 Mar 20	Lomela	Moderate	Primary
07–0288	11.0, M	NA	−	4	A	2007 Mar 20	Lomela	Moderate	Primary
07–0480	23.0, M	JX878427	−	4	A†	2007 May 25	Lomela	Severe	Primary
07–0514	7.0. M	JX878428	−	4	A†	2007 Jun 30	Lomela	Moderate	Primary
07–0623	12.0, M	NA	−	4	A	2007 Jul 15	Lomela	Moderate	Primary
07–0628	14.0, M	NA	−	4	A	2007 Jul 22	Lomela	Moderate	Secondary
07–0665	7.0, F	NA	−	4	A	2007 Sep 17	Lomela	Moderate	Secondary
07–0092	11.0, F	JX878415	−	5	A†	2006 Dec 26	Lomela	Moderate	Secondary
07–0045	14.0, F	JX878413	−	6	A†	2006 Dec 14	Lomela	Severe	Primary
07–0047	9.0, F	NA	−	6	A	2006 Dec 14	Lomela	Moderate	Secondary
07–0093	10.0, M	JX878416	−	6	A†	2006 Dec 26	Lomela	Moderate	Secondary
07–0094	7.0, F	NA	−	6	A	2006 Dec 26	Lomela	Moderate	Secondary
07–0126	13.0, F	NA	−	6	A	2007 Jan 17	Lomela	Moderate	Secondary
07–0286	20.0, M	JX878421	−	7	A†	2007 Mar 22	Lomela	Serious	Primary
07–0352	4.9, F	NA	−	7	A	2007 Apr 15	Lomela	Moderate	Secondary
07–0046	6.0, F	JX878414	−	8	A†	2006 Dec 14	Lomela	Moderate	Primary
07–0630	22.0, M	NA	−	10	A	2007 Jul 22	Lomela	NA	Secondary
06–0999	10.0, M	JX878409	−	13	A†	2006 Nov 9	Vangakete	Severe	NA
06–1070	3.4, M	JX878410	−	15	A†	2006 Nov 24	Vangakete	Moderate	NA
06–1075	5.0, M	JX878411	−	15	A†	2006 Nov 30	Vangakete	Severe	NA
06–1076	2.2, F	JX878412	−	14	A†	2006 Nov 30	Vangakete	Severe	NA
07–0121	15.0, M	NA	−	1	B	2007 Jan 3	Djalo-Ndjeka	Moderate	NA
07–0008	3.5, F	NA	−	2	B	2006 Dec 19	Ototo	Moderate	NA
07–0038	4.6, M	NA	−	2	B	2006 Nov 25	Lomela	Moderate	Primary
06–1009	21.0, M	NA	−	3	B	2006 Nov 13	Lomela	Moderate	Secondary
07–0039	0.9, F	NA	−	3	B	2006 Nov 25	Lomela	Moderate	Primary
07–0662	1.0, M	JX878429	−	5	B†	2007 Sep 4	Lomela	Severe	Primary
06–0950	2.2, F	JX878407	+	2	B†	2006 Oct 9	Kole	Serious	Secondary
07–0334	NA	NA	+	2	B	NA	Kole	NA	Secondary
07–0336	24.0, M	NA	+	2	B	2007 Mar 25	Kole	Mild	Primary
07–0338	3.8, M	JX878424	+	2	B†	2007 Mar 25	Kole	Mild	Secondary
07–0337	0.8, M	JX878423	+	3	B†	2007 Mar 25	Kole	Severe	Secondary
07–0339	20.0, F	NA	+	3	B	2007 Apr 2	Kole	Moderate	Secondary
07–0335	4.5, M	NA	+	4	B	2007 Mar 20	Kole	Mild	Secondary
07–0010	3.5, M	NA	+	6	B	2006 Dec 19	Ototo	Moderate	NA
07–0354	18.0, M	JX878425	+	7	B†	2007 Apr 20	Lomela	Moderate	Secondary
07–0035	1.8, M	NA	+	1	B	2006 Dec 24	Djalo-Ndjeka	Mild	NA
06–0970	10.0, F	JX878408	−	1	C†	2006 Oct 31	Katako Kombe	Serious	Primary
07–0120	0.4, F	JX878418	−	1	C†	2007 Jan 2	Djalo-Ndjeka	Severe	NA
07–0275	10.0, F	JX878419	−	1	C†	2007 Dec 10	Djalo-Ndjeka	Moderate	Primary
07–0276	1.5, M	NA	−	1	C	2007 Dec 10	Djalo-Ndjeka	Moderate	NA
07–0277	4.0, F	NA	−	1	C	2007 Dec 10	Djalo-Ndjeka	Severe	NA
07–0283	0.4, M	JX878420	−	1	C†	2007 Feb 26	Djalo-Ndjeka	Severe	NA
07–0450	11.0, M	JX878426	−	1	C†	2007 May 27	Kole	Severe	Secondary
07–0567	29.0, M	NA	−	1	C	2007 Aug 5	Vangakete	Severe	NA
07–0568	37.0, M	NA	−	1	C	2007 Aug 25	Vangakete	Moderate	NA
07–0610	4.3, M	NA	−	1	C	2007 Jul 23	Vangakete	Severe	NA
07–0104	4.0, F	JX878417	−	10	D†	2006 Dec 14	Bena-Dibele	Serious	Secondary

*Comprehensive overview of data obtained from sequencing and/or PCR analysis. Metadata are presented where available. Phylogenetic lineage (A–D) is defined for samples for which complete genomes were available. All other samples are grouped based on single-nucleotide polymorphism panel validation. The date listed is the date of sample collection. STR, short terminal repeat; NA, data point not available. †Full genome generated for this sample.

This genetic polymorphism was absent from all MPXV sequences available through GenBank. Moreover, although 1 full-length copy and 1 truncated copy of the OMCP gene are present in all genomes of the Western African clade MPXV, only the full-length copy of the gene is present in genomes of the Central African MPXV clade (Figure 2, panel A). No homologous gene is present in the VARV genome (*26*) (www.poxvirus.org). The deletion identified in our clinical samples completely removes the region of OMCP containing the leader sequence (Figure 2, panel B) and substantially alters protein folding (data not shown), suggesting that the truncated protein is nonfunctional. The deletion was identified in genomes belonging to lineage B only (Table). Data regarding route of infection were available for 44 patients; transmission for 24 cases was classified as human-to-human (secondary) and for 20 as animal-to-human (primary) (Table). Metadata were available for 8 of the samples containing the deletion; 7 (87.5%) of the 8 were from cases resulting from human-to-human transmission (p = 0.0544, Fisher exact test), suggesting that this deletion might be associated with increased fitness in humans. Further work, however, is needed to elucidate the role of OMCP in transmissibility.

### STR Analysis

 Substantial variations from other orthopoxviruses were detected in the 70-bp STR region (bases 72–486 and 196710–197124 in MPXV-COG_2003_358) (*4*,*32*–*34*). A traditional PCR designed against the left and right STR regions enabled us to evaluate this polymorphism in all 60 samples. We did not quantitatively test the purity of the repeat population beyond visualization of the PCR product by gel electrophoresis. A small percentage of the samples (10%) had faint secondary products that could not be resolved. The STRs described here should be considered to be the dominant population. In total, we identified 12 unique STR combinations (Table). STR variations have been described for VARV isolates obtained during 1944–1974 (*34*); however, no studies have addressed the possibility of STR variations in VARV isolates belonging to the same geographic cluster. Samples collected during episodes of VARV activity in Bangladesh, South Africa, or the Far East showed limited variation (<3 left STR patterns); whereas, we observed 12 patterns for MPXV samples collected during a 2-year period in the DRC. Given that the poxvirus genomes in our study were characterized directly from clinical samples, it is plausible that this smaller range of diversity for VARV is only the result of a genetic bottleneck that occurred during cell culture isolation. Rapid copy number variation of the K3L gene in the VACV ITR has recently been described as a mechanism for host immunomodulation (*25*). However, none of the STR copy number variations observed among the MPXV strains involved a known coding region. In contrast to some VARV isolates, all 60 MPXV samples had identical right and left STRs (Table).

### Phylogenetic Analysis of the Entire Dataset

 Because only 23 samples contained enough material for complete genome characterization, to expand our analysis to all 60 samples, we performed PCR amplification of a 410-bp variable region of the CRS corresponding to the H4L gene (VACV-COP gene notation). When selecting this region, we used the phylogenetic relationships of the CRS regions of the Central African clade by using 72 segregating sites over 61 kb of sequence. The selected region contained 4 segregating sites that are phylogenetically relevant to Central African clade MPXV (MPXV accession no. DQ01154: 89505, 89545, 89674, and 89914). No evidence of additional diversity was detected at these sites among the samples from the DRC except at the fourth site (89914) and only among the members of lineage B. Phylogenetic reconstruction of this region among the samples resulted in a topologic distribution for DRC isolates that correlates with the whole-genome tree (bootstrap values, as a measure for confidence measures, were lineage A (n = 12, 82%), lineage B (n = 11, 81%), lineage C (n = 6, 80%), and lineage D (n = 1). Moreover, this region contains 7 sites that differentiate between the Central African (n = 29) and Western African (n = 6) clades with a bootstrap value of 82%, which can be evaluated as another measure of the phylogenetically informative character of this genomic area. In this region, we identified 1 nonsynonymous and 3 synonymous changes. An additional nonsynonymous change was identified in all lineages ≈800 bp downstream of this region, for a total of 5 polymorphic sites in H4L within the Central African clade.

Overall, 11.6% of the genetic variations identified in this study were located in an ≈1.2-kb conserved region of the CRS. H4L is reported to be tightly associated with the DNA-dependent RNA polymerase, aiding in early gene transcription before initiation and termination (*35*,*36*). Increased mutation rates in a tightly constrained area of a genome can be a marker of increased selective pressure. The effect of the genetic polymorphisms on the function of H4L is unknown, and further studies to evaluate their effects on early gene expression are needed.

The phylogenetically informative single-nucleotide polymorphisms were assessed by PCR amplification and dideoxynucleotide sequencing for all 60 samples. This information was then used to extrapolate the lineage of each sample (Table). 

Lineage A contains the widest range of STR repeats (*1*–*15*) and represents 33 (55.0%) of the samples analyzed. The lineage seems to have arisen in the Lomela health zone (forest) where it continued to circulate throughout 2007. The remaining members of the lineage are a cluster of strains with 13–15 STR repeats that were geographically restricted to the Vangakete health zone (savannah). According to the World Health Organization scoring system for severity used during smallpox eradication, the virus strains belonging to lineage A caused predominantly moderate disease (66.7%, p = 0.2816). No significant differences in animal-acquired versus human-acquired infection were associated with this lineage.

Lineage B contains 1–8 STRs and represents 16 (26.7%) of the samples analyzed. These strains were predominantly isolated from the Kole (forest) and Lomela health zones and were associated with moderate disease (80.0%, p = 0.2179). Although no statistically significant association with secondary (human) transmission was observed among all lineage B strains, the MPV-Z-N2R/OMCP deletion was identified only in genomes belonging to this lineage and, as stated above, was significantly associated with human-to-human transmission.

Lineage C showed high homogeneity with only 1 STR and represented 10 (6.7%) of the samples analyzed. These strains were predominantly obtained from the Djalo-Ndjeka (ecotone) and Vangakete health zones and were associated with severe/serious disease (70.0%, p = 0.0234).

Only 1 representative of lineage D was identified and contained 10 STRs. The sample was collected in Bene Dibele (forest), was associated with severe disease, and was acquired from a human source.

Although the data do not support causality linking the number of STR repeats to the severity of any lineage, the composition of the STR region merits further investigation for effects on virulence and transmission and as a marker of passaging effects. Our data support a wide diversity of STR composition that varies according to lineage; 1 population exhibited only a dominating single STR repeat and others had variants between 1 and 15 repeats. On the basis of our data and the information previously reported (*25*) depicting an accordion-like expansion of gene-copy in response to antiviral systems, there is precedence for a copy number variation mechanism used by the virus. Considering that there is no coding element in this region, the most likely need to vary this region is structural; therefore, we predict that the STR region may be under selective pressure to provide stability to this region of the genome.

## Discussion

Our data demonstrate that a combination of genomic destabilization and genetic polymorphism are influencing the evolution of MPXV strains actively circulating in the Sankuru District of the DRC. Patterns of genomic destabilization and gene loss were consistent with models of orthopoxvirus genomic evolution (*7*). A recently submitted genomic sequence derived from 2 isolates obtained in Sudan in 2005 contains a large indel in the right flanking region. This indel simultaneously inserts a 10.8-kb inverted repeat of several immune modulatory coding regions from the left flanking region and deletes a 2.1-kb region that surrounds the region that we identified as reduced. The authors who reported the sequence posit that this insertion and deletion resulted from a single event, and the identifying sequence is the only evidence of either the insertion or the exact deletion in any *Poxviridae* isolate (*37*). The 2 reported changes cannot be functionally compared in this study; however, additional evidence supports genetic variability in this region. Genomic reduction might have played a role in the emergence of VARV as a highly adapted human pathogen capable of efficient human-to-human spread (*7*). Although it has been suggested that gene loss was associated with a restricted host range for VARV, no link has been demonstrated between gene loss and increased severity of disease. VARV is thought to have diverged from its rodent-borne ancestor 3,000–4,000 years ago (*38*). The strains of VARV isolated during periods of high activity in the 20th century were already well adapted to their evolutionary niche (humans), and, as a result, their genetic sequences were highly conserved. Conversely, the level of variability for MPXV isolates might suggest an active pattern of change; however, longer-term surveillance is required.

Although genomic changes are predicted by models of host transition, the correlation of the gene loss pattern with secondary transmission might indicate that MPXV is adapting for efficient replication in a novel ecologic niche: humans. It is also plausible that the association of OMCP gene loss and transmissibility is coincidental because numerous factors, including vaccination status and human encroachment on reservoir habitats, could also explain increased human-to-human transmission and variant introduction frequency. The scarcity of information regarding historical orthopoxvirus emergence and the absence of sequencing data for MPXV reservoir isolates precludes our ability to pinpoint the exact source of the observed variability; however, we predict that the 4 lineages are circulating in the reservoir population and are introduced into the human population after direct contact with those reservoir hosts. Further genomic surveillance that includes reservoir species might help explain the variant emergence described herein.

Active surveillance during November 2005–November 2007 in Sankuru District, DRC, suggested a strong correlation between smallpox vaccination and protection from MPXV (*20*). However, as the number of unvaccinated persons continues to increase, so does the size of the susceptible population. Other factors in the DRC, such as malnutrition, disease, and an inadequate health care system, have provided an ideal environment for MPXV. It is possible that increased circulation of MPXV in the human population of the DRC is a driving force in the evolution of the virus. Our data also indicate that certain genetic changes might affect disease severity or human-to-human transmissibility, suggesting an active period of adaptation that could result in virus strains with increased fitness in humans. However, we have no evidence to directly link the genetic changes to increased severity or transmissibility in vivo.

The global effects of the emergence of MPXV strains that are highly adapted to humans could be devastating. Importation of MPXV by infected vertebrates is of concern because of the potential for establishment of new reservoirs outside Africa. In fact, American ground squirrels have been found to be susceptible to infection (*39*), suggesting that other rodent species worldwide might also be susceptible. Small genetic changes could favor adaptation to a human host, and this potential is greatest for pathogens with moderate transmission rates (such as MPXV) (*40*). The ability to spread rapidly and efficiently from human to human could enhance spread by travelers to new regions. Therefore, active disease surveillance should continue to be used monitor MPXV for changes that are consistent with increasing adaptation to humans. Continued active surveillance of the Sankuru District, and expansion to all other regions where the virus is known or predicted to circulate, would help determine the true geographic range of this virus. Given the apparent rapid evolution of this virus, when suspected or confirmed cases in humans are observed, health authorities in presently unaffected areas should become vigilant and actively prepare to take immediate action.
